# Experience of the NPC Brazil Network with a Comprehensive Program for the Screening and Diagnosis of Niemann-Pick Disease Type C

**DOI:** 10.3390/ijns8030039

**Published:** 2022-06-28

**Authors:** Francyne Kubaski, Alberto Burlina, Giulia Polo, Danilo Pereira, Zackary M. Herbst, Camilo Silva, Franciele B. Trapp, Kristiane Michelin-Tirelli, Franciele F. Lopes, Maira G. Burin, Ana Carolina Brusius-Facchin, Alice B. O. Netto, Larissa Faqueti, Gabrielle D. Iop, Edina Poletto, Roberto Giugliani

**Affiliations:** 1 BioDiscovery Laboratory, Hospital de Clinicas de Porto Alegre, Porto Alegre 90035-903, Brazil; fkubaski@udel.edu (F.K.); afacchin@hcpa.edu.br (A.C.B.-F.); larissafaqueti@gmail.com (L.F.); gabrielle.iop@gmail.com (G.D.I.); 2 PPGBM, UFRGS, Porto Alegre 91501-970, Brazil; alicenetto@hcpa.edu.br (A.B.O.N.); edinapoletto@gmail.com (E.P.); 3 Medical Genetics Service, HCPA, Porto Alegre 90035-903, Brazil; francielebarbosatrapp@gmail.com (F.B.T.); ktirelli@hcpa.edu.br (K.M.-T.); franciele.f.lopes@gmail.com (F.F.L.); mburin@hcpa.edu.br (M.G.B.); 4 Division of Inherited Metabolic Diseases, Regional Center for Expanded Neonatal Screening, Department of Women and Child’s Health, University of Padova, 35129 Padova, Italy; alberto.burlina@unipd.it (A.B.); giulia.polo@gmail.com (G.P.); 5 Waters Technologies Do Brasil, São Paulo 06455-000, Brazil; danilopereira@innovatox.com.br (D.P.); camilo_silva@waters.com (C.S.); 6 Innovatox, São Paulo 18047-720, Brazil; 7 Department of Chemistry, University of Washington, Seattle, WA 98105-6250, USA; zherbst@uw.edu; 8 DASA, São Paulo 06455-010, Brazil; 9 INAGEMP, Porto Alegre 90035-003, Brazil

**Keywords:** Niemann-Pick disease type C, lysosphingomyelin-509, tandem mass spectrometry, biomarker, lysosomal storage disorder, Brazil

## Abstract

Niemann-Pick disease type C (NPC) is a lysosomal disorder caused by impaired cholesterol metabolism. Levels of lysosphingomyelin 509 (LysoSM509) have been shown elevated in dried blood spots (DBS) of NPC and acid sphingomyelinase deficiency patients. In this study, we report our experience using a two-tier approach (1st tier is the quantification of lysoSM509 by ultra-performance liquid chromatography tandem mass spectrometry followed by the 2nd tier with next-generation sequencing of the *NPC1* and *NPC2* genes). DBS samples from 450 suspected patients were received by the NPC Brazil network. Of these, 33 samples had elevated levels of lysoSM509, and in 25 of them, variants classified as pathogenic, likely pathogenic, or of unknown significance were identified in the *NPC1* or *NPC2* genes by next-generation sequencing. The quantification of lysoSM509 in DBS as a first-tier test for the diagnosis of NPC followed by molecular analysis of the *NPC1* and *NPC2* genes almost doubled the detection rate when compared to the performance of chitotriosidase activity as a first-tier biomarker, and it could likely be increased with the addition of a third tier with MLPA of the two genes involved. This strategy seems suitable for the neonatal screening (NBS) of NPC if this disease is eventually adopted by NBS programs.

## 1. Introduction

Niemann-Pick disease type C (NPC; OMIM#257220, 607625) is a neurodegenerative multisystem lysosomal storage disorder with an autosomal inheritance pattern caused by impaired intracellular lipid trafficking leading to the progressive accumulation of cholesterol, glycosphingolipids, phospholipids, and sphingomyelin due to pathogenic variants in the *NPC1* and *NPC2* genes, with 95% of the cases related to variants in the *NPC1* gene [1,2,3,4,5]. The storage can happen in a variety of organs and tissues, but the main organs affected are the liver, spleen, lungs, and the central nervous system (CNS) [5,6].

The main clinical features are hepatosplenomegaly, jaundice, fetal hydrops, hypotonia, ataxia, dysphagia, dystonia, and cognitive impairment, among other problems. [5,7] Due to the lysosomal storage of several of these lipids, especially cholesterol in the brain, NPC patients suffer from neurodegeneration due to the massive loss of Purkinje neurons in the cerebellum and diffuse atrophy in other regions, neuroinflammation, and demyelination [5,8,9]. The clinical spectrum is thus very heterogeneous, and it can be classified into four main groups based on the age of onset: early infantile, late infantile, juvenile, and adolescent/adult [10,11,12]; the lifespan of the patients can range from a few days of life to 60 years of age [7]. The estimated incidence of NPC is 1:100,000 to 120,000 [5].

Because of the unspecific symptomatology, the diagnosis of NPC can be challenging [6]. The Filipin staining was the first biochemical method employed for the diagnosis of NPC, and until recently it was considered the gold standard test for NPC diagnosis, but it is invasive, longstanding, and laborious [6,13]. Chitotriosidase is a biomarker that can be assayed in blood, and levels can be increased in NPC; however, this test is not specific for NPC as it can also be increased in other lysosomal and non-lysosomal disorders, or it can be normal in NPC patients, especially in late-onset forms [6,14]. Oxysterols have also been quantified in the plasma of NPC patients with marked elevated levels of cholestane-3β, 5α, 6β-triol (C-triol), and 7-ketocholesterol (7-KC) [15,16]. However, several patients have been reported without elevation or with borderline results for these markers. In addition, the biomarker is unstable and difficult to adapt to dried blood spots (DBS), limiting its usefulness, and there is also a limitation in the differential diagnosis of NPC from acid sphingomyelinase deficiency (ASMD) patients [6,16,17,18,19,20].

Another biomarker that has shed some light to improve the diagnostic odyssey of NPC patients is lysosphingomyelin-509 (LysoSM509, the carboxylated analog of lysosphingomyelin), which has been shown elevated in plasma and dried blood spots of NPC patients [21,22,23,24,25,26]. In this study, we report our strategy with a two-tier program for the high-risk screening and diagnosis of NPC using the quantification of LysoSM509 in DBS followed by confirmation with molecular analysis of the *NPC1* and *NPC2* genes in the same DBS sample, a strategy that can be potentially useful for neonatal screening.

## 2. Materials and Methods

### 2.1. Samples

DBS were collected from subjects with clinical suspicion of NPC from Bolivia, Brazil, Colombia, and Ecuador. All suspected patients were receiving no specific therapy at the sample collection. All tests were performed by the NPC Brazil network. DBS from 450 suspected individuals were analyzed and compared with 74 age-matched controls, and 30 general newborns. All samples were kept at −20 °C before the analysis. Ethical approval was obtained from the Ethics Committee of the HCPA (project #2019-0034). Informed consent was obtained from all subjects involved in the study. The data presented in this study are not publicly available due to ethical reasons but are available upon request to the corresponding author.

### 2.2. Chemicals and Reagents

Ultrapure formic acid, phenol, sodium acetate, and LC-MS grade organic solvents such as chloroform, 2-propanol, and methanol were purchased from Sigma Aldrich (Saint Louis, MO, USA). HPLC grade acetonitrile was purchased from JT Baker® (Radnor, PA, USA). Absolute ethanol was purchased from Merck (Darmstadt, Germany). Tris-HCL, EDTA, sodium chloride, and SDS were purchased from Neon (Suzano, Brazil). Proteinase K was purchased from Promega (Madison, WI, USA). Ultrapure water was obtained from the Milli-Q system from Millipore (Bedford, NY, USA), and for the molecular assays ultrapure DNase/RNase-free distilled water was purchased from ThermoFisher Scientific (Waltham, MA, USA).

Since no isotopic labeled internal standard is currently commercially available for the analysis of lysoSM509, lysosphingomyelin-d7 (lysoSM-d7) was used as the internal standard. LysoSM-d7 was purchased from Avanti Polar lipids (Alabaster, AL, USA). The working solution was 2.5 nmoL/L of lysoSM-d7 according to the method previously described [25,26].

### 2.3. Sample Analyses

#### 2.3.1. Quantification of LysoSM509

One 3.2mm disc of DBS was incubated with 100 µL of 2.5 nmoL/L of lysoSM-d7 in a 96-well polypropylene plate at 45 °C at 500 RPM for 1 h. After the incubation, the plate was centrifuged for 5 min at 3000 G. The supernatant was transferred to a new 96-well plate and 50 µL of MilliQ H_2_O was added [26]. Then, 10 µL were injected into the ultra-performance liquid chromatography tandem mass spectrometer (UPLC-MS/MS) [26]. The mass spectrometer was a Xevo TQ-S micro from Waters (Milford, CT, USA) with an XSelect® CSH™ C18 3.5 µm (2.1 × 50 mm) from Waters. The column was kept at 55 °C.

The mobile phase was a gradient elution of 70:30 H_2_O with 0.1% formic acid [solution a] to 65:35 isopropanol/acetonitrile with 0.1% formic acid [solution b]. The flow rate was 0.8 mL with a gradient of: 99.5% A at 0 min, 75% A at 0.75 min, 40% solution A at 1 min, 25% solution A at 1.5 min, 100% solution B at 1.80 min, 100% solution B at 2.15 min, 99.5% solution A at 2.20 min. The mass spec was operated with electrospray on positive mode with multiple reaction monitoring (MRM). Precursor and product ions (*m*/*z*) were used to quantify lysoSM509 509,184; lysoSM-d7 472.4,184 with 30 V for cone and 22 V for collision. The injection volume was 10 µL per sample with a running time of 2.20 min. The levels of lysoSM509 were expressed as nmoL/L.

#### 2.3.2. Chitotriosidase Enzyme Activity

The Chitotriosidase enzyme activity was quantified by fluorimetry following the protocol previously described [27]. The reference range is 0–44 nmoL/h/mL.

#### 2.3.3. Molecular Analysis

DNA was extracted from 5 3.3 mm discs using an in-house protocol. In summary, the protocol starts with the incubation of the DBS spots with the extraction buffer and proteinase K at 56 °C to break down the membranes. Then, phenol is used to clean the proteins, followed by the use of chloroform to purify the aqueous phase. After that, the precipitation of the aqueous phase is performed with salt and absolute ethanol. In the end, the pellet is washed with 70% ethanol, dried, and resuspended in distilled H_2_O. After the extraction, the DNA was quantified using the Qubit^®^double stranded DNA (dsDNA) High sensitivity (HS) assay kit (Invitrogen) (Waltham, MA, USA) to obtain the concentration data.

Post-extraction, the DNA samples were analyzed through next-generation sequencing (NGS) using the Ion S5 System platform (Thermo Scientific, Waltham, MA, USA) with a customized panel (Ion AmpliSeq™Thermo Scientific, Waltham, MA, USA) including the *NPC1* and *NPC2* genes. The data were analyzed on Ion Torrent suite and Ion reporter (Thermo Scientific, Waltham, MA, USA) version 5.0. The reference sequences used were NM_000271.4 and NM_006432.3.

#### 2.3.4. Statistical Analysis

GraphPad Prism v.8.0 was used for the statistical analyses. Datasets were not normally distributed according to the normality and log normality tests: Anderson–Darling test, D’Agostino and Person, Shapiro–Wilk, and Kolmogorov–Smirnov tests. The Kurskal–Wallis with Dunn’s post-hoc test was used to compare lysoSM509 levels in suspected NPC patients, age-matched controls, and general newborns at the level of significance of 0.05. Pearson’s correlation with a 95% confidence interval was used to verify the correlation between the levels of lysoSM509 and Chitotriosidase activity.

## 3. Results

A total of 450 DBS from patients with suspected NPC from Latin America (mostly from Brazil) were received and were compared with 74 age-matched controls and 32 general newborns. A total of 33 samples had elevated levels of lysoSM509 (Table 1).

In the 33 subjects with increased lysoSM509 levels, the mean age was 8.8 years of age (range: 2 months to 53.8 years of age), and 54% were females (Table 1). The mean age for the healthy controls was 12.3 years of age (range: 1 month to 69.7 years of age).

**Figure 1:**
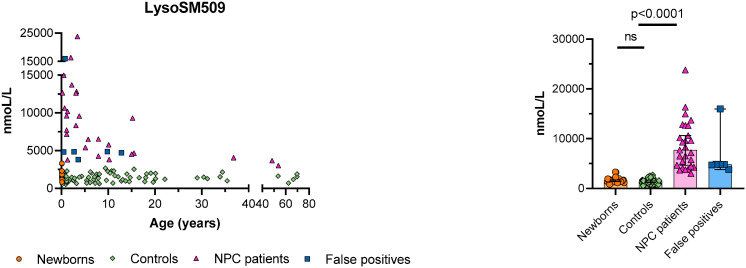
Distribution of lysoSM509 in general newborns, age-matched controls, NPC patients, and “false positives” (samples with normal NGS of the *NPC1* or *NPC2* genes, but without MLPA of the *NPC1* and *NPC2* genes). Medians with a 95% confidence interval are shown in the grouped plot.

The mean level of lysoSM509 in the 33 subjects who screened positive was 8318 nmoL/L (range: 3003–23,812 nmoL/L). The mean level of lysoSM509 in the controls was 1399 nmoL/L (range: 535–2672 nmoL/L). The mean level of lysoSM509 in the general newborns was 1587 nmoL/L (range: 838–2311 nmoL/L). The mean level of lysoSM509 was significantly higher in the subjects who screened positive compared to general newborns and controls (*p* < 0.0001) (Figure 1). The samples with high LysoSM509 with normal NGS of the *NPC1* and *NPC2* genes without a molecular diagnosis were defined as “false positives” (Figure 1).

**Figure 2:**
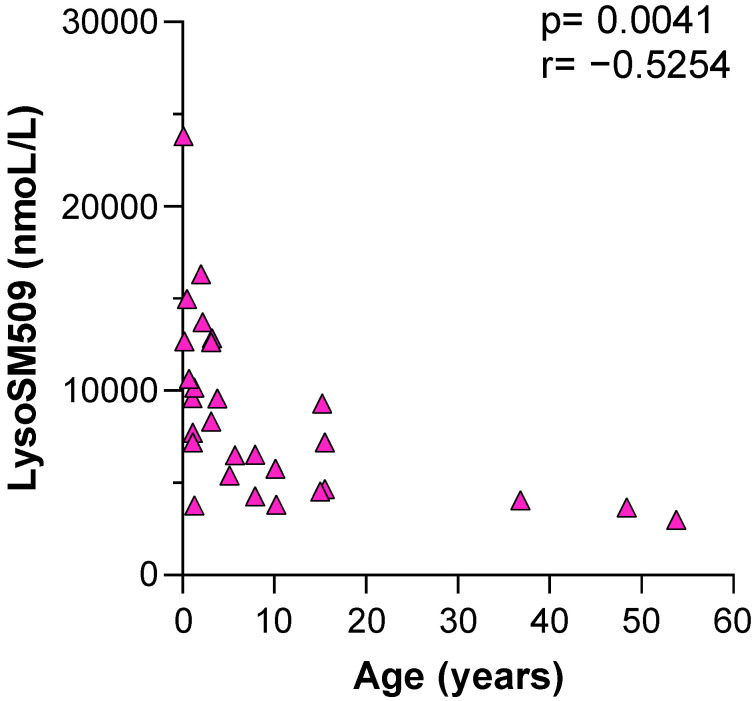
Pearson’s correlation of the lysoSM509 level × age in the possible NPC patients.

Pearson’s correlation coefficient was used to check if there was a correlation between the levels of lysoSM509 and age. There was a negative correlation observed with a Pearson’s correlation coefficient of −0.5254 (*p* = 0.0041) (Figure 2). All samples with elevated levels of lysoSM509 and available chitotriosidase results were included in this analysis (with or without confirmation by NGS) (Figure 2).

Chitotriosidase activity levels were available for 31 out of the 33 samples with elevated levels of lysoSM509 (Table 1). The mean activity of Chitotriosidase was 50 (range: undetectable-309 nmoL/h/mL) (reference range = 0–44 nmoL/h/mL). Out of these 31 samples, 12 (39%) had elevated Chitotriosidase activity. Four samples (13%) had undetectable levels of Chitotriosidase activity, and sequencing of the *CHIT1* gene revealed they were all compound heterozygotes for two novel variants: p.Trp358Ter (rs3831317; classified as benign by the American College of Medical Genetics-ACMG), and p.Ala359Gly (rs201682373; classified as a variant of unknown significance (VUS) by the ACMG).

**Figure 3:**
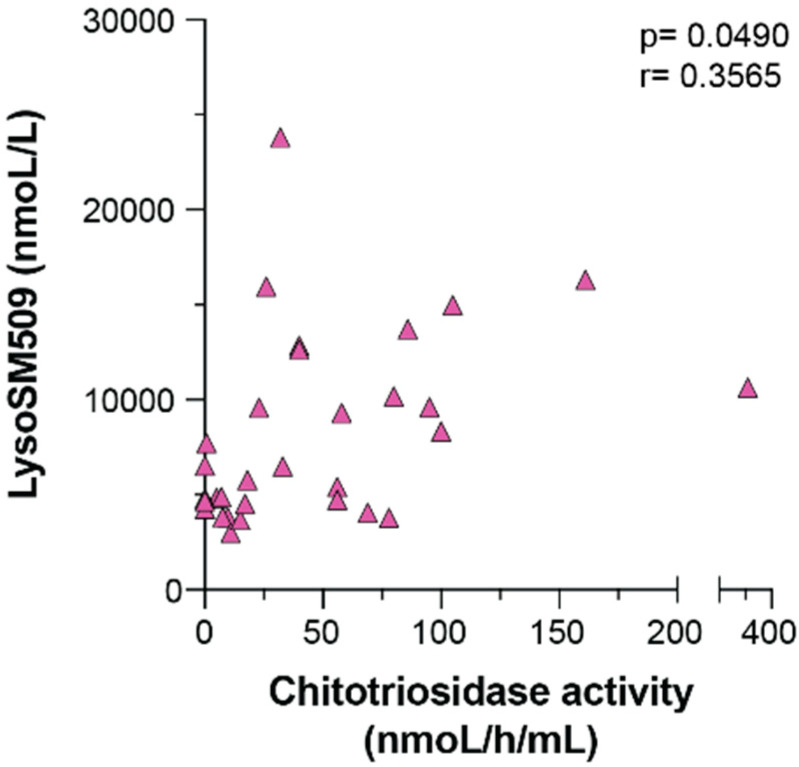
Pearson’s correlation between the activity of Chitotriosidase activity compared with the levels of lysoSM509.

Pearson’s correlation coefficient was used to check if there was a correlation between the activity of Chitotriosidase and lysoSM509. A positive correlation was observed with a Pearson’s correlation coefficient of 0.3565 (*p* = 0.0490) (Figure 3).

**Figure 4:**
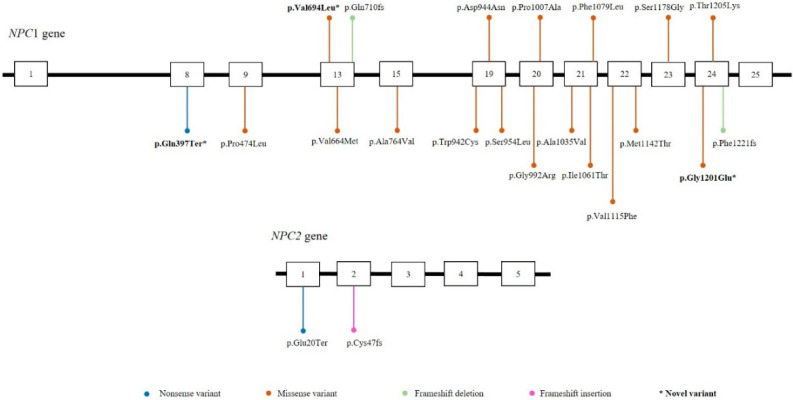
Schematic representation of the distribution of the exonic variants in the *NPC1* and *NPC2* genes. Boxes represent exons of the *NPC1* and *NPC2* genes. * Novel variants identified.

As part of our two-tier high-risk screening program, all of the samples with elevated levels of lysoSM509 were analyzed by NGS of the *NPC1* and the *NPC2* genes. Pathogenic variants were found in 79% of the suspected patients confirming the NPC diagnosis (Table 1). Pathogenic variants were identified throughout the length of the *NPC1* and the *NPC2* genes with no particular hotspot regions (Figure 4). To our knowledge, three variants were reported for the first time in Brazilian patients, and they were classified as pathogenic and likely pathogenic the ACMG, respectively (p.Gln397Ter, p.Val649Leu, and p.Gly1201Glu) (Figure 4, Table 1).

A total of 27 out of the 33 samples (82%) had pathogenic variants in the *NPC1/NPC2* genes. In total, 94% of the pathogenic alleles identified were in the *NPC1* gene, and the most frequent variant was p.Ala1035Val in 16 alleles (four homozygous patients and eight heterozygous) (Table 1). Six samples (18%) had no pathogenic variant identified in the *NPC1* or *NPC2* genes by NGS (described as “false positives” in Figure 1) (Table 1), and in two patients (6%) only one pathogenic variant was identified by NGS (Table 1). It is not possible to rule out the NPC diagnosis in these cases without having additional molecular analyses performed, such as multiplex ligation-dependent probe amplification (MLPA), which were not available. Furthermore, we have not measured the levels of acid sphingomyelinase (ASM) in those samples, but we have measured the levels of lysoSM and all of them had normal levels, likely excluding the diagnosis of ASMD. Thus, from the 450 DBS received we were able to diagnose 25 NPC patients, with eight further patients still needing additional molecular analysis.

## 4. Discussion

NPC is a metabolic neurodegenerative disorder with a heterogeneous presentation that can often delay the diagnosis or, not rarely, lead to misdiagnosis [1]. Several therapeutic options have been explored or are under development for NPC. Substrate inhibition therapy with Miglustat was already approved in several countries. Currently, clinical trials are exploring the benefits of the combination therapy of anti-inflammatory coupled with Miglustat, 2-hydroxypropyl-β-cyclodextrin (HP-β-CD) alone or combined with Miglustat, Vorinostat, Arimoclomol, as well as gene therapy [28]. As in most conditions with neurodegeneration, early diagnosis and early treatment are instrumental to provide better benefits to affected patients [29].

The quantification of biomarkers such as sphingolipids by tandem mass spectrometry has improved the diagnosis of NPC patients, reducing the need to perform invasive skin biopsies for the Filipin test (which is performed in growing fibroblasts). Several studies have shown the elevation of lysoSM509 in NPC patients [21,22,24,25,30]. Based on these findings, we decided to start a two-tier program with the quantification of lysoSM509 by UPLC-MS/MS coupled with NGS of the *NPC1* and *NPC2* genes as part of the diagnostic workflow of the NPC Brazil Network to provide accurate and faster screening/diagnosis to Latin American NPC patients. It is also important to mention that because of the long distances and delays with regular mail, it was a major landmark to be able to provide the diagnosis using solely DBS that can be shipped at room temperature throughout our large continent.

We have shown that the quantification of lysoSM509 in DBS is a reliable marker for the diagnosis of NPC (*p* < 0.0001) (Figure 1, Table 1), that the levels of lysoSM509 have a negative correlation with age (*p* = 0.0041) (Figure 2), and a positive correlation with the levels of Chitotriosidase activity (*p* = 0.0490) (Figure 3). Interestingly, only 39% of the samples with increased lysoSM509 levels had elevated activity of chitotriosidase, indicating the low yield of chitotriosidase as a biomarker for screening (Table 1). In addition, 13% of the samples were not informative regarding Chitotriosidase activity due to variants in the *CHIT1* gene. This well-documented problem affects the use of Chitotriosidase for NPC screening. It is unclear at this time if lysoSM509 is also actually elevated in NPC patients in the newborn period. No newborn samples from NPC patients were available to us, and it is important that future pilot studies elucidate if lysoSM509 can be a valuable marker for NBS of NPC if this condition becomes a target for NBS.

With NGS performed in the 33 samples that presented increased lysoSM509 levels, we were able to identify 25 patients with NPC. However, we were limited by the fact that only NGS of the *NPC1* and *NPC2* genes were available at our lab, and thus we could not exclude NPC in the eight samples with elevated levels of lysoSM509 without pathogenic variants identified by NGS (Table 1, Figure 1). A possible reason for a normal molecular result through NGS in samples with elevated levels of lysoSM509 could be due to the limitation of the NGS technique in detecting copy number variants (large deletions and insertions). For these cases, the most appropriate technique would be the multiplex ligation-dependent probe amplification (MLPA) [6,29,31]. MLPA, RNA, and protein analysis would potentially contribute to elucidating the cause of lysoSM509 elevation in the eight cases without variants identified in the *NPC1* and *NPC2* genes, but it was not possible for us to perform this extended molecular analysis at this time. Another limitation of this study was the larger amount of sample required for DNA extraction. If this method is included in our nationwide NBS, we would need to further optimize the DNA extraction with a lower sample volume (three or one DBS punches instead of five).

The genetic identification of NPC can be complex; until this moment, 575 pathogenic variants have been identified in the *NPC1* gene and 30 in the *NPC2* [32]. Three novel variants (p.Gln397Ter, p.Val649Leu, and p.Gly1201Glu) were identified in Brazilian patients (Figure 4, Table 1), and to our knowledge, they have not been described in the literature. This highlights the fact that several NPC patients have private variants.

## 5. Conclusions

Before the validation of the lysoSM509, the NPC Brazil Network was analyzing solely the levels of Chitotriosidase activity, performing molecular analysis of the *NPC1* and *NPC2* genes in the samples with increased Chitotriosidase activity. As shown in this study, Chitotriosidase is not a reliable screening biomarker. If solely this assay had been used as a first-tier, we would miss the diagnosis of 14 NPC patients (56%) (Table 1). Our two-tier high-risk screening program (quantification of lysoSM509 followed by NGS of the *NPC1* and *NPC2* genes) showed an impressive diagnostic rate. The addition of a third-tier with molecular analysis of selected cases by multiplex ligation-dependent probe amplification (MLPA) and potentially other methods would further improve its performance. Provided appropriate reference values are well established in newborns (as we demonstrated, they are age-dependent), this approach using DBS could be scaled up to be suitable for neonatal screening when NPC becomes an NBS target. However, we were limited by the fact that no newborn NPC samples were available at the time of this study to demonstrate elevation. Pilot studies are needed to confirm the suitability of this method for NBS of NPC, and to further elucidate the disease incidence, only by having pilot studies we can confirm the sensitivity and specificity of this method as a tool for NBS. Nonetheless, in the advent of NBS expansion by multiplex methods with UPLC-MS/MS, this method can be multiplexed with the assay of several lysosomal enzymes with a short running time of less than 3 min per sample. It is also important to mention that currently no approved therapies exist for the treatment of asymptomatic NPC patients, although there are several clinical trials with new drugs in progress. In our opinion, having a screening protocol established, when such therapies are approved and available, may lead to the consideration of this disease to be included in NBS programs.

## Figures and Tables

**Table 1:** Demographics of the possible NPC patients with biochemical and molecular results.

ID	Gender	Age at Sample Collection †	Country	LysoSM509 (nmoL/L) ^1^	Chitotriosidase Activity (nmoL/h/mL) ^2^	Genotype	Gene with Variant(s)
1	Male	3.11	Brazil	8328	100	p.A1035V/p.A1035V	*NPC1*
2	Female	1.11	Brazil	7722	0.8	p.Q710fs/p.Q710fs	*NPC1*
3	Female	1.1	Brazil	9609	95	p.A1035V/p.A1035V	*NPC1*
4	Female	10.2	Brazil	3819	9.7	p.A1035V/p.V694L	*NPC1*
5	Male	1.4	Brazil	10,169	80	p.F1221fs/p.A1035V	*NPC1*
6	Male	10.10	Brazil	5773	18	p.C47fs/-	*NPC2*
7	Female	36.8	Brazil	4049	69	p.A1035V/p.G992R	*NPC1*
8	Male	3.6	Brazil	3814	7.6	-/-	?
9	Male	5.11	Brazil	5401	56	p.A764V/p.A764V	*NPC1*
10 *	Male	8.9	Brazil	4271	#	p.T1205K/p.P1007A	*NPC1*
11 *	Male	8.9	Brazil	6521	#	p.T1205K/p.P1007A	*NPC1*
12	Male	5.7	Brazil	6495	33	p.A1035V/-	*NPC1*
13	Female	2	Brazil	16,323	161	p.W942C/p.W942C	*NPC1*
14	Male	2.7	Brazil	4839	5	-/-	?
15	Female	0.7	Brazil	10,632	309	p.A1035V/p.Q397 *	*NPC1*
16	Male	0.8	Brazil	15,958	26	-/-	?
17	Female	3.8	Brazil	9571	23	p.F1221fs/p.A1035V	*NPC1*
18	Male	0.5	Brazil	4820	#	-/-	?
19	Female	2.2	Brazil	13,706	86	p.W942C/p.W942C	*NPC1*
20	Female	1.3	Brazil	3785	78	p.F1079L/p.A1035V	*NPC1*
21	Female	3.4	Brazil	23,812	32	p.F1221fs/p.I1061T	*NPC1*
22	Female	3.2	Brazil	12,834	40	p.A1035V/p.G1201E	*NPC1*
23	Female	0.2	Brazil	12,662	n/a	p.A1035V/p.A1035V	*NPC1*
24	Female	53.8	Brazil	3003	11	p.S954L/p.P474L	*NPC1*
25	Female	1.11	Brazil	7180	n/a	p.A1035V/p.A1035V	*NPC1*
26	Male	15.5	Brazil	4669	#	p.A1035V/p.A1035V	*NPC1*
27	Male	48.4	Brazil	3672	15	p.P1007A/p.P1007A	*NPC1*
28	Male	3.1	Colombia	12,628	40	p.D944N/p.D944N	*NPC1*
29	Female	0.5	Colombia	14,980	105	p.E20 */p.E20 *	*NPC2*
30	Male	15	Bolivia	4525	17	p.S1178G/p.V1115F	*NPC1*
31	Female	15.2	Colombia	9310	58	p.M1142T/p.V1664M	*NPC1*
32	Female	9.8	Colombia	4881	7	-/-	?
33	Female	12.8	Colombia	4726	56	-/-	?

† Age when DBS samples were collected in untreated patients; * Twins; #: undetectable levels of Chitotriosidase activity; n/a: not available; -: no variants found in that specific allele by NGS; ^1^ Reference Range (controls): 525–2672 nmoL/L; ^2^ Reference range (controls): 0–44 nmoL/h/mL; ?: no variants identified in the *NPC1* or *NPC2* genes.

## Data Availability

Data presented in this study are available on request from the corresponding author. The data are not publicly available due to data protection restrictions.
